# Genomics Reveal Population Structure and Intergeneric Hybridization in an Endangered South American Bird: Implications for Management and Conservation

**DOI:** 10.1002/ece3.70820

**Published:** 2025-01-08

**Authors:** Marisol Domínguez, Larissa S. Arantes, Pablo D. Lavinia, Nicole Bergjürgen, Agustin I. Casale, Pablo A. Fracas, Darío A. Lijtmaer, Pablo Tubaro, Sarah Sparmann, Susan Mbedi, Camila Mazzoni, Bettina Mahler, Ralph Tiedemann

**Affiliations:** ^1^ Unit of Evolutionary Biology/Systematic Zoology Institute for Biochemistry and Biology, University of Potsdam Potsdam Germany; ^2^ Department of Evolutionary Genetics Leibniz Institute for Zoo‐ and Wildlife Research (IZW) Berlin Germany; ^3^ Berlin Center for Genomics in Biodiversity Research (BeGenDiv) Berlin Germany; ^4^ Laboratorio de Investigación y Conservación de la Biodiversidad (UNRN‐InCoBIO) Universidad Nacional de Río Negro Viedma Argentina; ^5^ Universidad Nacional de Río Negro, CIT Río Negro (UNRN‐CONICET) Viedma Argentina; ^6^ Museo Argentino de Ciencias Naturales “Bernardino Rivadavia” (MACN‐CONICET) Buenos Aires Argentina; ^7^ Leibniz Institute of Freshwater Ecology and Inland Fisheries (IGB) Berlin Germany; ^8^ Museum für Naturkunde ‐ Leibniz Institute for Evolution and Biodiversity Science Berlin Germany; ^9^ IEGEBA, FCEN‐UBA, Pabellón II Ciudad Universitaria Buenos Aires Argentina

**Keywords:** 3RADseq, fluidigm, genomic assignment, introgression

## Abstract

Genomics is an invaluable tool for conservation, particularly for endangered species impacted by wildlife trafficking. This study uses genomic data to provide new insights to aid conservation and management of endangered species, using as a case study the Yellow cardinal ( *Gubernatrix cristata* ), a bird endemic to southern South America severely affected by illegal trade and the transformation of its natural habitat. We explore population structure within the Yellow cardinal, delimiting management units and describing connectivity among them. Additionally, we develop and assess the accuracy of a panel of 189 informative SNPs, and demonstrate how these can reliably assign confiscated individuals to one of the management units established. Lastly, we assess hybridization between the Yellow cardinal and the Diuca finch ( *Diuca diuca* ), which is reported to occur in regions of sympatry. We confirm that hybridization occurs, although it is not as common as previously thought, and that hybrids might be fertile, as we found evidence of backcrossing with Yellow cardinals. We discuss the implications of this introgression for the evolution and conservation of Yellow cardinals. Our study provides new, valuable information that can guide conservation efforts, comprising a test case for the use of genomics in combating illegal trafficking, with potential application beyond the case of the Yellow cardinal.

## Graphical abstract

This study uses genomics to aid conservation of the endangered Yellow cardinal ( *Gubernatrix cristata* ), impacted by illegal trade, habitat loss, and potentially hybridization with the Diuca finch ( *Diuca diuca* ). It identifies management units, tracks confiscated individuals using a small SNP panel, and confirms occasional hybridization. Our findings offer valuable insights for conservation and combating wildlife trafficking, with broader applications beyond the Yellow cardinal.

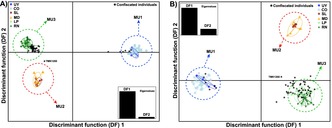

## 1 Introduction

The unprecedented population declines in global biodiversity highlight the urgent need to understand, monitor, and develop effective conservation strategies (Ceballos et al. 2015). Conservation genomics emerges as a powerful tool to address these challenges by assessing conservation status, monitoring genetic diversity, delineating conservation units within species, combating illegal wildlife trafficking, and unraveling complex conservation issues such as hybridization. By integrating genetic evidence, more targeted and informed strategies can be designed to preserve and restore biodiversity, ensuring the long‐term survival of threatened species.

The wildlife trade poses a significant threat to the conservation of many species (Carpenter et al. 2005; Butchart et al. 2010; Brook et al. 2012). In particular, the illegal capture and commercialization of wild birds not only reduce their population sizes but also disrupt the natural genetic diversity and structure of the species. Here, conservation genomics plays a crucial role particularly by providing tools to monitor genetic diversity, identify population structure, and trace the geographic origin of confiscated individuals. These genetic insights are essential for informing reintroduction and translocation efforts, ensuring that individuals are returned to appropriate habitats that will maximize their chances of survival and reproduction. Moreover, genomics can help classify management units, which are vital for implementing targeted conservation strategies. Information derived from genetic studies can also shed light on hybridization patterns, revealing how widespread and common hybridization is among species and its potential impact on genetic integrity.

Understanding the impact of hybridization is essential to protect threatened species. While reproductive isolation mechanisms and natural selection often prevent the formation of animal hybrid lineages (Ottenburghs 2018), hybridization still plays a significant role in bird speciation. This is evidenced by the existence of species of hybrid origin, such as the Italian sparrow ( *Passer italiae* , Hermansen et al. 2014), and the golden‐crowned manakin ( *Lepidothrix vilasboasi* , Barrera‐Guzman et al. 2017), as well as avian hybrid zones, such as those between hooded and carrion crows ( *Corvus cornix* and *Corvus corone* , respectively, Poelstra et al. 2014), and pied and collared flycatchers ( *Ficedula hypoleuca* and *Ficedula albicollis* , Burri et al. 2015). Introgressive hybridization, where genetic material from a species is incorporated into the gene pool of another one through backcrossing, can have significant conservation implications (Arnold 2006). Hybridization can potentially lead to: (1) the establishment of a stable and localized hybrid zone that does not lead to the disappearance of the original parental species; (2) the merging of the two parental species into one, resulting in a single, genetically blended population; or (3) the appearance of a new species (hybrid speciation, Mallet 2007), which is distinct from both parent species and capable of reproducing independently. In threatened species, hybridization can impact effective population size and genetic integrity (Rhymer 2008). Identifying and understanding such genetic exchanges across species borders is crucial for developing conservation strategies that preserve the unique genetic makeup of endangered species.

**FIGURE 1:**
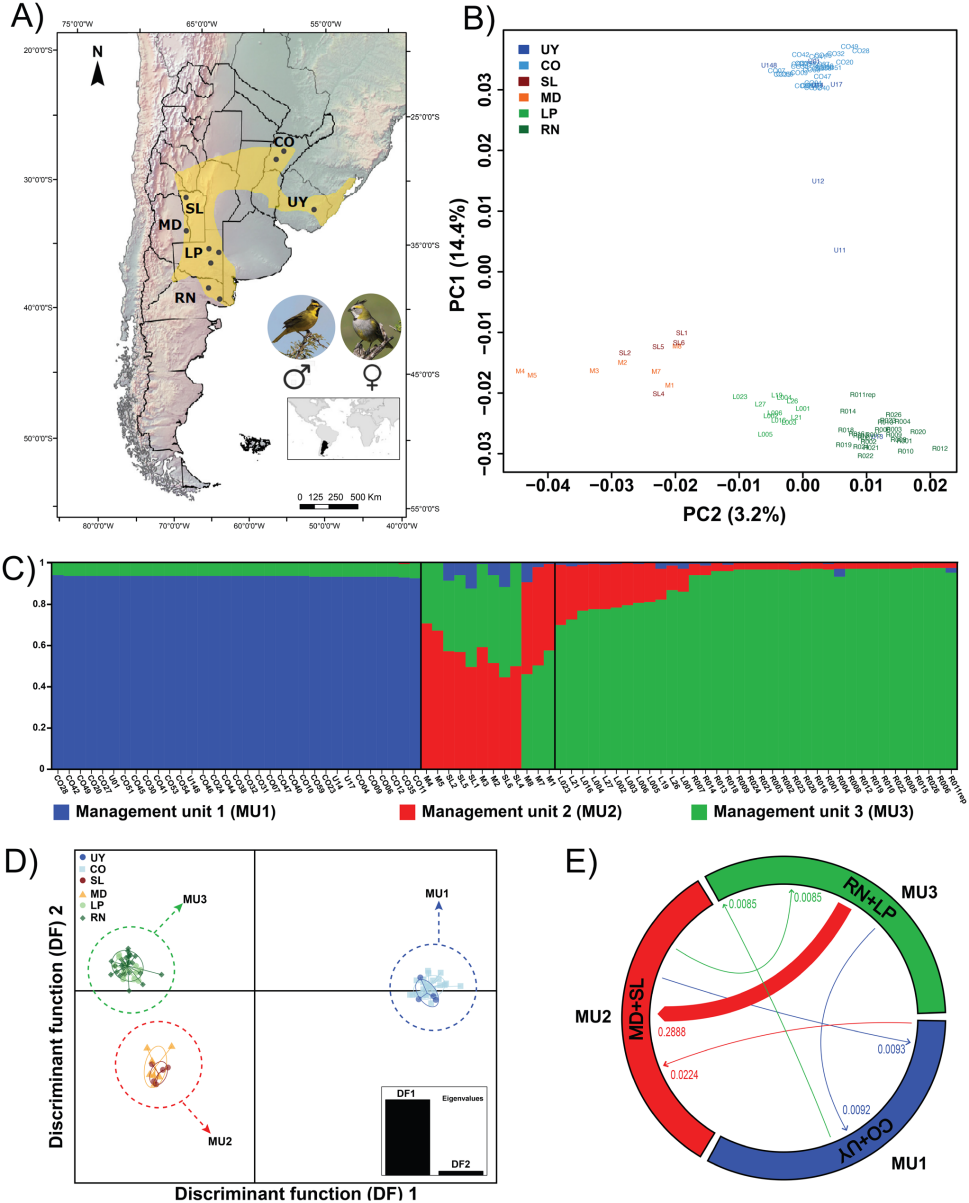
Population structure within Yellow cardinals. (A) Map showing the historical distribution of the Yellow cardinal and the sampling localities (gray points): Uruguay (UY), and the Argentinian provinces of Corrientes (CO), San Luis (SL), Mendoza (MD), La Pampa (LP), Rio Negro (RN). (B) Principal coordinates analysis based on Nei distances with samples colored by sampling location. (C) Structure plot (*K* = 3). (D) Scatterplot of the discriminant analysis of principal components (DAPC) for *K* = 3. (E) Inferred contemporary migration rates between genetic clusters. Numbers refer to the means of the posterior distributions of *m*, the migration rate into each population. Arrows show the direction of the migration, from the source to the receiving population.

The Yellow cardinal ( *Gubernatrix cristata* ) is a sexually dimorphic, monotypic passerine bird endemic to southern South America, that constitutes a prime example of the wildlife trade effects and a paradigm for avian conservation in the region. Historically, the species had a continuous distribution and was associated with dry and thorny shrublands, woodlands and other savanna‐like environments of Argentina, Uruguay and Brazil (Figure 1A). However, habitat loss related to the fragmentation of its main habitat, the Espinal woodland, due to wood extraction and agriculture, resulted in its current, highly fragmented distribution with its main populations being found in Argentina (Domínguez et al. 2021). At the same time, the Yellow cardinal is the most sought after species by wild bird trappers (Argibay, Dabul, and Diaz 2005; Pessino and Tittarelli 2006; Loydi 2008). For over a century, this species has suffered continuous removal of individuals, mostly males (Pessino 2006), to stock the illegal cage‐bird market (Pessino and Tittarelli 2006). All of this together has led to a marked reduction in population size, resulting in its current classification as an endangered species, with an estimated population size of less than 2000 individuals (BirdLife International 2024a). A previous genetic study based on microsatellite markers identified three genetically distinct management units (MUs), revealing a strong east–west split and a lower differentiation between the southern and northern populations to the west (Domínguez et al. 2017). Nevertheless, the absence of further population structure in addition to these three main genetic groups could either reflect high gene flow within the MUs or could be a result of the limitations in the analytical power of a small panel of microsatellites to detect spatial structuring.

The Yellow cardinal hybridizes with its sister species, the Diuca finch ( *Diuca diuca* ; Burns et al. 2014; Barker et al. 2015), in the southern region of its distribution where they coexist during the reproductive season (Bertonatti and Lopez‐Guerra 1997; Pessino 2006). The Diuca finch is a polytypic and partially migratory species that ranges from southern Bolivia and northern Chile to southern Patagonia, and from sea level to 2000 m. In comparison to the Yellow cardinal, the Diuca finch appears as a less ecologically constrained species, having a much wider distribution and inhabiting a more diverse array of environments, from Espinal woodlands and Monte shrublands to the Patagonian Steppe and forest edges. This results in areas of both sympatry and allopatry between the two species (BirdLife International 2024b). Even though hybrids with the Yellow Cardinal have been observed in the areas of sympatry (Bertonatti and Lopez‐Guerra 1997; Pessino 2006), their reproductive behavior and whether they are fertile is unknown. In terms of phenotype, hybrids are morphologically similar to the Yellow Cardinal but exhibit a plumage coloration more typical of the Diuca Finch overall: their upperparts are generally gray with a black crown and crest, the breast and flanks are gray, and it has a white supercilium, belly and malar area bordering a black throat (Pessino 2006). Moreover, the hybrid has 18 remiges like the Diuca finch, while the Yellow Cardinal has 16 (Bertonatti and Lopez‐Guerra 1997).

Here we use RAD‐seq data to explore broad‐ and fine‐scale population structure across the range of Yellow cardinals, and to investigate the impact of hybridization on the genetic pattern of the threatened species. From the general perspective of the maintenance of species boundaries, it is important to understand how far isolation between Yellow cardinals and Diuca finches depends on mate availability, and whether the absence of conspecific mating partners could blur species limits in this system. At the same time, a proper understanding of how introgression could affect cardinal populations is key for the species' conservation. In this context, we: (1) describe population structure and genomic diversity of Yellow cardinals' populations, (2) develop and assess the accuracy of a panel of informative SNP markers, and assign the most probable population of origin of confiscated cardinals, and (3) investigate whether hybridization and introgression between species actually occur and, if so, how frequent and pervasive it is. Finally, we discuss the implications of our results to the evolution, management and conservation of Yellow cardinal populations.

## 2 Materials and Methods

### 2.1 Sampling

We sampled a total of 86 wild Yellow cardinals covering most of their entire known range, including all predefined management units (Domínguez et al. 2017) and a new location previously not studied (Figure 1A, Table S1). We also included 48 blood samples from confiscated Yellow cardinals from the illegal wildlife traffic that were provided by Argentinian national authorities. In the case of the Diuca finch, we sampled a total of 31 individuals from the subspecies *D. d. minor*, focusing on the region of sympatry with the Yellow cardinal (Table S1). Lastly, we included samples from three individuals that were putatively classified as hybrids based on their phenotype (Table S1). Field work was conducted with the authorizations of the corresponding *Direcciones de Fauna*, which granted all collection permits needed.

Genomic DNA was isolated using the DNeasy Tissue Extraction Kit (Qiagen, Hilden, Germany) from blood and tissue samples, and used to obtain genomic data by Restriction site‐associated DNA Sequencing (RAD‐Seq), to amplify the mitochondrial gene cytochrome c oxidase subunit I (COI), and for molecular sex determination of the putative hybrid individuals.

### 2.2 Library Construction

We used the 3RADseq method (Bayona‐Vásquez et al. 2019) with some modifications (Hoffberg et al. 2016) to build the genomic libraries for all sampled individuals. First, the DNA underwent digestion using EcoRI, MspI, and ClaI restriction enzymes (New England Biolabs). The first two enzymes generate sticky ends to facilitate adapter ligation, while ClaI was utilized to cleave adapter‐dimers formed by phosphorylated adapters. A total of 200 ng of DNA with 10 U/μL of each enzyme and a unique pair of barcodes were incubated at 37°C for 1 h. After digestion, DNA ligase and ATP were added to the reaction, which was then exposed to multiple temperature cycles to promote ligation (22°C) followed by digestion (37°C), ending with 80°C for 20 min. Barcoded samples were equimolarly pooled and subjected to fragment size selection, targeting fragments between approximately 230–420 bp, using a BluePippin system equipped with a 1.5% cassette and the R2 marker (Sage Science). This range was chosen aiming to select around 32,600 loci, as estimated by the *in silico* digestion analysis of the *G. cristata* reference genome (GenBank accession number JBEIAG000000000). The 40 μL size‐selected product was split into four aliquots and each replicate underwent a single‐cycle PCR to incorporate the iTru5‐8N primer, followed by an 8‐cycles indexing PCR using P5 outer and P7 primers. The iTru5‐8N index contains 8 degenerate bases that uniquely tag initial DNA molecules and allow the identification and removal of PCR duplicates during preprocessing of the sequencing data (Hoffberg et al. 2016). The quality of final libraries was assessed using the Agilent Tapestation system. A small‐scale sequencing (around 8000 reads per individual) was performed to screen samples for endogenous DNA content and check the balance between individuals in a sequencing pool. The proportion of reads belonging to each individual was used to calculate new volumes of the digestion/ligation product that were then re‐pooled for a second library preparation, aiming for an equal representation of each individual in the final library. This weighted re‐pooling strategy is described by Arantes et al. (2023). Final libraries were sequenced using 150 bp paired‐end reads on a partial lane of the NovaSeq S4 platform (Illumina), targeting a minimum coverage of 30× per individual.

### 2.3 Preprocessing Analysis and SNP Calling

The initial preprocessing step was the adapter trimming of the raw reads using the software Cutadapt (Martin 2011). Data were then demultiplexed per individual based on the dual‐internal barcodes using Flexbar software (Roehr, Dieterich, and Reinert 2017). PCR duplicates were removed using a custom Python script (https://git.imp.fu‐berlin.de/begendiv/radseq‐preprocessing‐pipeline/‐/blob/main/scripts/filterPCRdups/filterPCRdups_CM.py), which identifies identical reads with matching iTru5‐8N index sequences and keeps only one copy of the original DNA molecule. Paired‐end reads were then merged using PEAR v.0.9.11 (Zhang et al. 2014) setting the maximum length of the assembled sequences to 230 bp, aiming to filter out the short fragments. Unassembled forward and reverse reads were trimmed to the maximum length of 130 bp and minimum quality of 30 using the Trimmomatic software (Bolger, Lohse, and Usadel 2014). Then, a custom Python script (https://git.imp.fu‐berlin.de/begendiv/radseq‐preprocessing‐pipeline/‐/blob/main/scripts/digestion/checkRestrictionSites.py) was used to check for restriction sites, retaining only reads with correct sequences at both ends. Finally, undigested and chimeric sequences were removed by removing any sequence with internal restriction sites. Individuals exceeding 4.1 million reads were subsampled to ensure even coverage across individuals.

Filtered reads were mapped against the reference genome of *G. cristata* using Bowtie v2.3.5.1 (Langmead and Salzberg 2012) with default parameters and the flags “‐no‐mixed” and “‐no‐discordant.” Sex scaffolds were identified using SatSuma v3.1.0 (Grabherr et al. 2010) and removed from posterior analysis. The resulting sorted bam files were used as input in the Stacks reference‐based pipeline v2.61 (Catchen et al. 2011), with which we performed the loci building and SNP calling to create four different datasets. The first dataset (YC_raw), which consisted of 84 Yellow cardinals of known origin from six different locations, was used for preliminary exploration of genomic grouping. After these exploratory analyses, three individuals were excluded (see Section 3), resulting in the second dataset (YC) of 81 wild Yellow cardinals of known origin, which was used for further analyses. The third dataset (YCC) included these 81 Yellow cardinals plus 48 confiscated individuals, and the fourth (YCDH) consisted of 81 wild cardinals, 27 wild Diuca finches and three putative hybrids.

For the YCDH dataset, the Populations pipeline from Stacks was run allowing only loci genotyped in at least 60% of individuals in a species and present in all three groups to be retained in the final dataset (*p* = 3 and *r* = 0.6). For the other datasets, only loci genotyped in at least 60% of individuals within at least one group to be retained in the final dataset (*p* = 1 and *r* = 0.6). To obtain a set of unlinked SNPs for population structure analysis, the data was restricted to the first SNP per locus (–write‐single‐snp). Further variant filtering was performed on the VCF file using VCFtools (Danecek et al. 2011). We progressively filtered out variants with low (< 10×) and high (two times the average coverage) coverage, and high percentage of missing data (allowing a maximum of 20%). The number of SNPs remaining after each filter and the final number of SNPs for each dataset are presented in Table S2. We estimated the pairwise kinship with PLINK v2.0—make‐king‐table option and excluded one member of each pair of samples with kinship coefficient greater than 0.1, which corresponds to second‐degree relations (Purcell et al. 2007). The clean datasets were used for different downstream analyses, as described below.

### 2.4 Population Structure and Genomic Diversity

SNP data management and genomic diversity analyses were performed in R‐4.3.0 (R Core Team 2019) using wrapper functions of the R package SambaR v1.10 (https://github.com/mennodejong1986/SambaR, de Jong et al. 2021). We first investigated the global population structure of wild endangered Yellow cardinals (dataset YC_raw) by performing a principal coordinate analysis (PCoA) using the function “pcoa” of the R package ape‐5.8 (Paradis and Schliep 2019) on Nei's genetic distance matrices, as it is an assumptions free approach which allowed us to visualize the genetic variation among Yellow cardinals.

Further assessment of genetic structure within Yellow cardinals was based on the YC dataset. We first used STRUCTURE 2.3.4 (Pritchard, Stephens, and Donnelly 2000) to run 10 replicates for each value of *K* from 1 to 5 under the admixture ancestry model and with correlated allele frequencies. Each run was done as 500,000 generations following a burn‐in of 200,000 generations. The most likely *K* was assessed with both Evanno's *ΔK* method (Evanno, Regnaut, and Goudet 2005) and Puechmaille's four estimators (Puechmaille 2016) as implemented in StructureSelector (Li and Liu 2018). Runs for the same *K* values were combined in clumpp 1.1.2 (Jakobsson and Rosenberg 2007) and plotted with STRUCTURE PLOT 2.0 (Ramasamy et al. 2014). To further explore genetic structure within the species, we ran fineRADstructure (Malinsky et al. 2018), which is based on previous software developed by Lawson et al. (2012). We ran the program under default parameters, retaining a maximum of 10 SNPs per RAD locus and allowing no more than 45% missing data per individual. This haplotype‐level approach combines all SNPs from each RAD locus to obtain a co‐ancestry matrix. The dataset consisted of 23,522 RAD loci and a total of 133,813 SNPs, and results were plotted in R using the fineRADstructurePlot.R and FinestructureLibrary.R scripts provided with the package (https://www.milan‐malinsky.org/fineradstructure). Finally, we performed a discriminant analysis of principal components (DAPC) in *adegenet* v.2.1.10 (Jombart 2008) to visually assess differentiation between genomic clusters. In contrast to a principal component analysis, the DAPC maximizes the separation between groups while minimizing variation within them, resulting in clearer separation between clusters (Jombart, Devillard, and Balloux 2010). We defined clusters a priori based on STRUCTURE and fineRADstructure results, and used the cross‐validation method to establish the number of principal components to be retained. For cross‐validation, we ran 30 replicates using 90% of the individuals as training dataset and the remaining 10% as the validation set.

To quantify the levels of genetic differentiation between the clusters recovered from the analyses described above, we calculated pairwise genome‐wide *F*_ST_ estimates (Weir and Cockerham 1984) using the function “stamppFst” of the R package StAMPP‐1.6.3 (Pembleton, Cogan, and Forster 2013) with 1000 permutations. Genome‐wide diversity within clusters was estimated with the function “calcdiversity” of the R package SambaR.

### 2.5 Gene Flow Estimation

To estimate the intensity and direction of contemporary gene flow between management units, we employed BayesAss BA3‐SNPs (Wilson and Rannala 2003; Mussmann et al. 2019). This software uses a Bayesian approach coupled with Markov Chain Monte Carlo (MCMC) to infer posterior probability distributions of individual immigrant ancestries. We analyzed the YC dataset comprising 1581 unlinked SNPs with no missing data (Table S2). Prior to analysis, we optimized the mixing parameters to achieve an acceptance rate between 20% and 60%, as recommended by the developers of the program. Specifically, we set the migration rate, allele frequency, and inbreeding coefficient to 0.6. The MCMC was run for 50,000,000 iterations, discarding the initial 5000,000 as burn‐in, and sampling every 5000 iterations. Convergence of the MCMC chains was assessed using TRACER v. 1.7.2 (Rambaut et al. 2018), with a minimum effective sample size (ESS) threshold of 200 considered sufficient for parameter estimation. The migration rate is presented in a circos plot created with the R package Circlize v.0.4.15 (Gu et al. 2014).

### 2.6 Genomic Population Assignment of Illegally Traded Birds

We used *adegenet* to assign confiscated samples to the genomic clusters found within the Yellow cardinal. We first divided the YCC dataset (Table S2) into wild individuals of known origin (81) and confiscated samples (48). We then repeated the DAPC analysis described above for the wild cardinals only and used the function *predict.dapc* to predict the position of the seized cardinals onto the discriminant functions obtained. We obtained posterior membership probabilities for all individuals and plotted the results in a DAPC scatterplot. We then explored different combinations of diagnostic SNPs that could allow future assignment of confiscated cardinals of unknown origin to the genetically distinct MUs recovered here. To design this reduced panel of SNPs, we used VCFtools to estimate per‐site *F*_ST_ values among MUs and ranked the positions from highest to lowest in terms of differentiation (negative values were interpreted as zero). From a total of 269,913 SNPs, we retained 18 SNPs with *F*_ST_ ≥ 0.80 to capture differentiation between MUs 1 and 3, 15 SNPs with *F*_ST_ ≥ 0.90 for MU1 vs. MU2 clusters, and 159 SNPs with *F*_ST_ ≥ 0.55 to distinguish MU2 from MU3, the two least differentiated genomic clusters. This resulted in a dataset of 189 outlier SNPs (three positions were shared among comparisons) which was used in further tests (Table S3).

To assess the assignment efficacy of this SNP panel, we first used principal components analyses and Monte Carlo cross‐validation procedures as implemented in the assignpop v1.3.0 package in R (Chen et al. 2018) to split the wild cardinals of known origin into training and test datasets. We built a predictive model using a support vector machine (model svm) classification based on training sets composed of the most informative 50% loci and a random sample of 50% of individuals in the data set. The remaining 50% of individuals were used to test the rate of assignment, which was then averaged across 50 iterations. Finally, we repeated the DAPC analysis described above to assign the 48 seized individuals to the MUs based on these 189 outlier SNPs, and then compared the results with those obtained with the complete set of SNPs.

### 2.7 Hybridization

We tested if the putative hybrids are indeed true hybrids, or if they have been misdiagnosed. Using the YCDH dataset with 111 individuals (including hybrids) and 14,327 SNPs (Table S2), we first explored the major axes of genetic diversity among species and putative hybrids running a PCoA based on Nei distances and calculating genome wide heterozygosity for each sample using SambaR functions *ape_pcoa* and *calcdiversity*, respectively. We also ran STRUCTURE for K2 with the same settings detailed before to identify the genomic composition of the putative hybrids and assess admixture between parental species.

We estimated a genomic hybrid index with R package *gghybrid* version 0.0.0.9000 (Bailey 2023), which uses a Bayesian algorithm to calculate the proportion of alleles inherited from parental references. We assigned reference parental populations as *Gubernatrix cristata* (HI = 0) and *Diuca diuca* (HI = 1). A total of 20,000 MCMC iterations were performed with the first 5000 discarded as burn‐in.

Ancestry‐informative SNPs were identified as sites with fixed allelic differences between parental species. We expected F1 hybrids to be almost exclusively heterozygous at these specific loci, while backcrosses should lead to a skew in the distribution of genotype frequencies. We used VCFtools to estimate per‐site *F*_ST_ values between species allowing no missing data. This resulted in a set of 133 SNPs (*F*_ST_ = 1) that was used to assign individuals to parental species and hybrid categories using NewHybrids v.1.1Beta3 (Anderson and Thompson 2002). The analysis was done using a burn‐in period of 10,000 followed by 50,000 MCMC iterations with Jeffrey option and no priors. Results were plotted using the R package HybridDetective v0.1.0.90 (Wringe et al. 2017).

We amplified 694 bp near the 5′ end of the cytochrome c oxidase subunit I (COI) mitochondrial gene to reveal the identity of the maternal species, using the pair of primers BIRDF1 (Hebert et al. 2004; 5′‐TTCTCCAACCACAAAGACATTGGCAC‐3′) and COIBIRDR2 (Kerr et al. 2009; 5′‐ACGTGGGAGATAATTCCAAATCCTGG‐3′). Polymerase chain reaction (PCR) reagents and conditions can be found in Table S4. Sequencing was performed bidirectionally in an ABI 3500 Sanger Sequencer at the University of Potsdam, Germany, with the same primers used for amplification at the University of Potsdam, Germany. Sequences were edited using BioEdit v7.2.5 (Hall 1999). We obtained sequences for 12 yellow cardinals (representing all genetic clusters found with genomic data), 9 Diuca finches, the 3 putative hybrids, and the Red‐crested ( *Paroaria coronata* ) and Yellow‐billed ( *Paroaria capitata* ) cardinals to be used as outgroups (see Table S1 for GenBank accession numbers). We inferred a gene tree based on Bayesian methodology using MrBayes 3.2.2 (Ronquist et al. 2012). The best‐fitting model of nucleotide evolution was HKY + I based on the Bayesian information criterion (BIC) as implemented in jModelTest 2.1.1 (Darriba et al. 2012). We conducted two independent runs of 10 million generations under default priors and sampling trees every 100 generations. The average standard deviation of split frequencies between runs was < 0.01, indicating convergence. We used TRACER to confirm that both runs reached stationarity and that we had a good sample of the posterior probability distribution. After discarding the first 25% of sampled trees as burn‐in, the remaining 75,000 topologies of each run were combined to generate a 50% majority rule consensus tree.

Mitochondrial sequences were also used to estimate genetic distances between and within species in MEGA (Tamura et al. 2011). We then applied the standard molecular avian clock (2.1% sequence divergence per million years; Weir and Schluter 2008) to estimate divergence time between the Yellow cardinal and the Diuca finch. It has been shown that COI evolutionary rate is more constant (i.e., clock‐like) in comparison to other mitochondrial genes, making it a reliable choice for molecular dating (Lavinia et al. 2016).

Molecular sex determination of the putative hybrids was performed using primers P2 (5′ TCT GCA TCG CTA AAT CCT TT 3′) and P8 (5′ CTC CCA AGG ATG AGR AAY TG 3′) of Griffiths et al. (1998) to amplify intron fragments of the gen CHD1 gene. PCR conditions are detailed in Table S5. PCR products were separated electrophoretically on a 2% agarose gel.

## 3 Results

### 3.1 Population Structure and Genomic Diversity

The clustering of individuals in the PCoA obtained from genomic data clearly correlates with the geographic distribution of sampling localities (Figure 1B). The first principal coordinate accounted for 14.4% of the total genetic variation, separating individuals from the east and the west, while the second principal coordinate explained 3.2% of the total genetic variance and separated individuals from the center and the south of Argentina. Three individuals from Uruguay (UY) showed an unexpected grouping pattern: U13 clustered with individuals from Río Negro (RN), while U11 and U12 were placed between western and eastern localities. If human‐mediated dispersal is common, U13 could be a cardinal originally from RN that was released in Uruguay, while U11 and U12 might be descendants of a cross between western cardinals relocated to Uruguay and local individuals. These three samples were excluded from further analysis, as we consider they do not reflect natural migration events but rather human‐mediated dispersal.

Bayesian analyses of population structure revealed the existence of different genomic clusters within the Yellow cardinal. While the best number of clusters was 2 according to Evanno's Δ*K* method (Figure S1), the *K* value with the highest likelihood was 3 (Figure S2), which was further supported by all four Puechmaille's estimators as the optimal number of genomic clusters (Figure S3). The fineRADstructure analysis also supported the existence of these three main groups, suggesting some further substructure within each of them at the same time (Figure S4). Consequently, we retained *K* = 3 for further analyses, in agreement with previous studies (Domínguez et al. 2017). We named the three genomic clusters (Figure 1C) as management units (MU): MU1 (blue) including individuals sampled in Uruguay (UY) and Corrientes (CO) province (Argentina), MU2 (red) composed of individuals from Mendoza (MD) and San Luis (SL) provinces (Argentina), and MU3 (green) grouping cardinals from La Pampa (LP) and Rio Negro (RN) provinces (Argentina). Even though five individuals (SL4, SL6, M1, M7, and M8) showed membership coefficients close to 50/50 between MU2 and MU3, we decided to include them within the former as they all clustered within MU2 in all other analyses. MU1 is the most differentiated genomic cluster (Table 1, Figure 1D) and exhibits the lowest genomic diversity (Table S6). The geographically more proximate MU2 and MU3 are genetically much closer (Table 1) and exhibit clear signs of admixture (Figure 1A,C). Consistently, migration rate estimates (Figure 1E) showed that a large number of individuals in MU2 (San Luis and Mendoza) are migrants from MU3 (Rio Negro and La Pampa). Migration rate estimates between other MUs are comparatively much lower.

**TABLE 1:** Genomic differentiation between Yellow cardinal's genetic clusters.

	MU1	MU2	MU3
MU1	—		
MU2	0.080	—	
MU3	0.086	0.025	—

*Note:* Weir and Cockerham 1984 pairwise *F*_ST_ values. All values are significant at *p* < 0.001. MU = management unit.

### 3.2 Genomic Population Assignment of Illegally Traded Birds

Self‐assignment tests demonstrated that the panel of 189 candidate SNPs was as effective as the complete set of SNPs in discriminating the population of origin of samples of known origin (Table 2). MU1 and MU3 exhibited a self‐assignment accuracy of 100% irrespective of the SNP data set implemented. MU2 exhibited a lower assignment accuracy (66%) with all SNPs, which was however strongly increased to 92% with the panel of diagnostic SNPs (Table 2).

**TABLE 2:** Assignment accuracy. Self‐assignment tests were performed using a Monte‐Carlo cross‐validation method between the genetic clusters.

(A)			
Origin	Assignment (189 SNPs)		
	MU1	MU2	MU3
MU1	1.00 ± 0.00		
MU2	0.00 ± 0.00	0.92 ± 0.10	0.08 ± 0.10
MU3	0.00 ± 0.00	0.00 ± 0.00	1.00 ± 0.00

(B)			
Origin	Assignment (12,591 SNPs)		
	MU1	MU2	MU3
MU1	1.00 ± 0.00		
MU2	0.00 ± 0.00	0.66 ± 0.21	0.34 ± 0.21
MU3	0.00 ± 0.00	0.00 ± 0.00	1.00 ± 0.00

**FIGURE 2:**
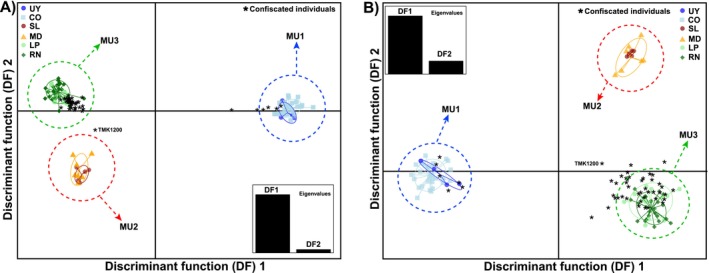
Genomic assignment of illegally traded yellow cardinals. (A) DAPC scatterplot for *K* = 3 and the complete set of unlinked SNPs. (B) DAPC scatterplot for *K* = 3 using the selected reduced set of 189 diagnostic SNPs.

Each of the seized cardinals was assigned with maximum posterior membership probability (1.0) to one of the three MUs. With the complete set of SNPs, 85.4% (41 out of 48) of confiscated birds were assigned to MU3, only one (TMK1200) to MU2, and 6 (12.5%) to MU1 (Figure 2A). Results were almost identical with the reduced set of 189 diagnostic SNPs (Figure 2B), with the only difference that TMK1200 was in this case assigned to MU3 (42 out of 48 confiscated samples, 87.5%).

### 3.3 Hybridization

**FIGURE 3:**
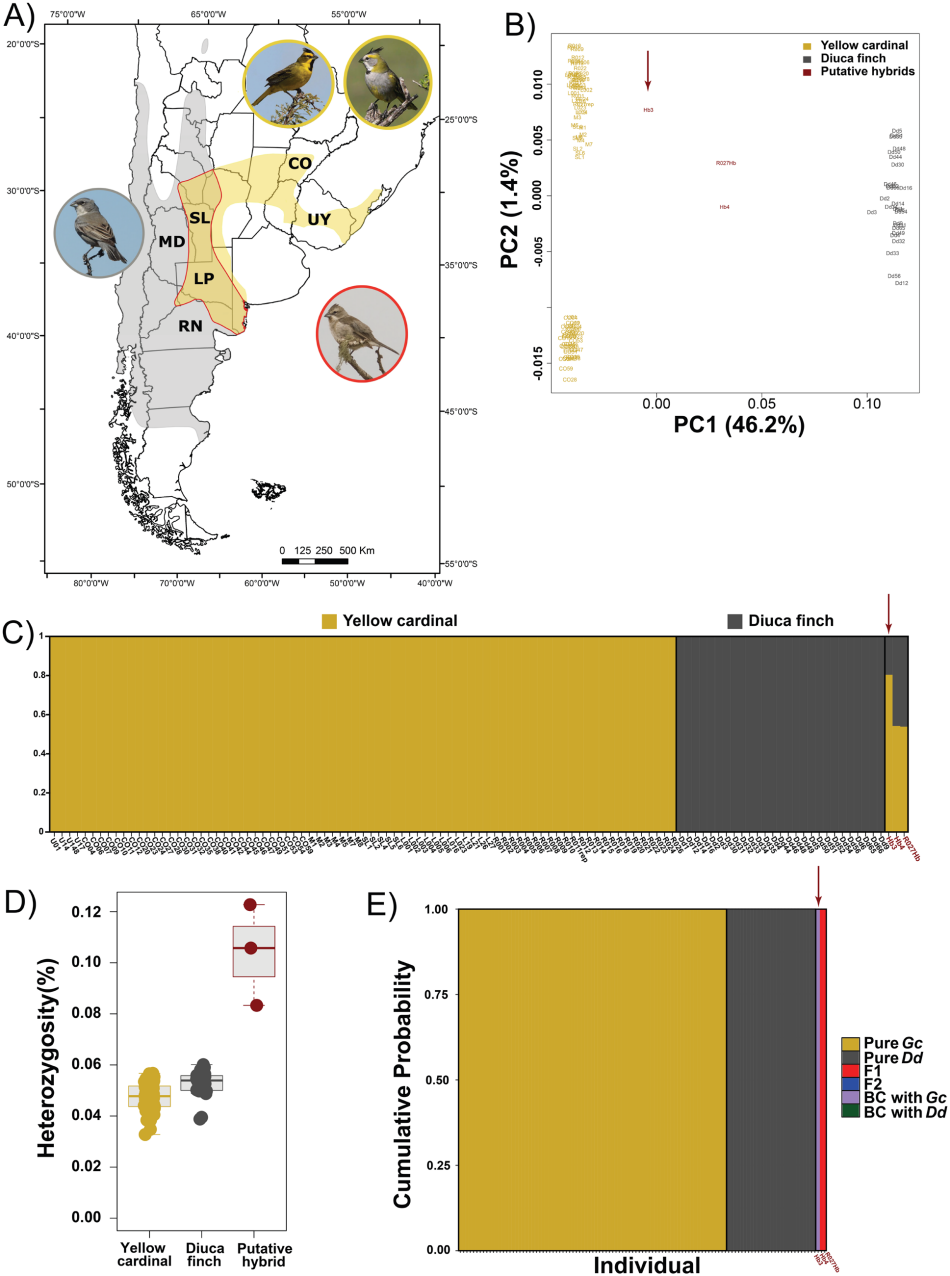
Hybridization between Yellow cardinals and Diuca finches. (A) Distribution map for both species highlighting the region of sympatry with a red border, and photos of Yellow cardinals (male and female, yellow border), a Diuca finch (gray border) and a putative hybrid (red border). (B) Principal coordinates analysis based on Nei distances and 14,327 unlinked SNPs. (C) Structure plot *K* = 2 based on 14,327 SNPs. (D) Genome‐wide heterozygosity based on 14,327 SNPs. (E) Hybrid categories (F1: Red; backcrossed with Yellow cardinal: Violet) assignment based on 133 fixed SNPs. The arrow in panels B, C, and E indicates the individual Hb3, identified as a backcross with Yellow cardinal. Photo credits: Elvira Canio for the Diuca finch and Carlos Figuerero for the other pictures.

Putative hybrid individuals were similar to Yellow cardinals based on their morphological characteristics, but similar to Diuca finches according to their plumage coloration (Figure 3A). The placement of these individuals was intermediate between the parental species in the PCoA (Figure 3B), suggesting that they could indeed be true hybrids and prompting us to conduct further analysis. The STRUCTURE analysis for *K* = 2 (Figure 3C) showed a lack of admixture between species and revealed a nearly equal contribution of each parental cluster for two of the three putative hybrids, and greater contribution from the Yellow cardinal in the third one (Hb3). Finally, these three individuals exhibited higher genome‐wide heterozygosity (Figure 3D).

In accordance with the STRUCTURE analysis, pure and hybrid classifications among *G. cristata* and *D. diuca* were well supported by the estimated hybrid index (HI) (Figure S5). Based on 133 SNPs fixed (*F*_ST_ = 1) between parental species (Table S7), we found that two of the three putative hybrids are heterozygotes at nearly all of these positions, while Hb3 exhibits ~60% of cardinal genotypes and ~ 30% of heterozygous positions, a result that is consistent with that of STRUCTURE and supports that this individual is not an F1 hybrid. The results for the different class assignments showed 100% posterior probability support for all individuals being pure cardinals or pure Diucas, except for the two F1 hybrids and Hb3, which was identified as a backcross with Yellow cardinal (Figure 3E).

The Bayesian phylogenetic tree based on the mitochondrial COI gene recovered both parental species as reciprocally monophyletic lineages with maximum support (Figure S6). Mean genetic divergence between the Yellow cardinal and Diuca finch was 8.1% (min 7.8%–max 8.5%), suggesting a Pliocene split around 3.85 Mya (range 3.71–4.05 Mya). The three hybrids clustered within the Yellow cardinal lineage, indicating that hybridization events occurred between female Yellow cardinals and male Diuca finches or male F1 hybrids. The molecular sex determination revealed that all hybrids were males.

## 4 Discussion

In this study, we used nuclear genomic (3RADseq) data to investigate population structure within the Yellow cardinal, an endangered bird species from southern South America, that is currently threatened by illegal trafficking, environmental loss and, maybe, introgression with the Diuca finch in regions of sympatry. We also propose the delimitation of management units and incentivize the use of a reduced set of nucleotides that will aid in the future assignment of confiscated individuals to their putative place of origin. Finally, we discuss the implications of hybridization between these species, and provide remarks on future evolutionary and conservation studies for this system.

### 4.1 Delimitation of Management Units and Its Application on Illegal Wildlife Traffic

Understanding population genetic structure is essential for designing conservation efforts to ensure successful management (Funk et al. 2012). Our study confirmed the existence of three genomic clusters or management units (MUs) within the Yellow cardinal, with a deep phylogeographic break between the east (where MU1 is located) and west (MU2, MU3) areas of the Yellow cardinal distribution. This separation is probably due to barriers to gene flow, as indicated by the lower migration rates and highest *F*_ST_ values between MU1 to the east and MU2 and MU3 to the west. This split could be related to the existence of an ecological barrier associated with the contrasting environmental characteristics of the regions inhabited by eastern (more humid) and western (more dry) populations (Oyarzabal et al. 2018). Further, the Paraná‐Paraguay river axis in *Del Plata* River basin could have also acted as a dispersion barrier between eastern and western populations. This river has been suggested as a geographical barrier for other bird species as well (Brodkorb 1939; Short 1975; Hayes 1995; Campagna et al. 2014; Kopuchian et al. 2020). Conversely, approximately 30% of individuals in MU2 were inferred as migrants originating from MU3. This high migration rate from MU3 into MU2 implies habitat continuity in this region and a potential larger population size in MU3, which might explain the admixture detected between both MUs in the Bayesian structure analyses. Migration rates between MU1 and western MUs are very low, further suggesting these are isolated populations. Moreover, lower genomic diversity in MU1 has significant conservation consequences. The loss of genetic diversity may leave the population vulnerable to novel environmental conditions, such as those resulting from climate change. This vulnerability highlights the urgent need for targeted conservation strategies to maintain genetic diversity within MU1.

The three MUs reported here constitute a pattern similar to that found by Domínguez et al. (2017) using nuclear microsatellites and fewer geographic locations. However, the greater number of SNPs from this study has revealed a clearer genetic differentiation (i.e., higher membership coefficients) that enabled us to develop an efficient panel of SNPs that can successfully discriminate among populations. For this, we isolated the most informative markers (highest *F*_ST_ estimates between the three MUs) to genotype future confiscated individuals using a relatively inexpensive and high‐throughput SNP genotyping approach. We designed the SNP panel with future management applications in mind, such as Fluidigm SNP genotyping (Wang et al. 2009). Due to the dependency on 96‐well plates of the Fluidigm EP1 system, we aimed at identifying fewer than 192 SNPs to compose the panel. The proposed panel of 189 unique markers proved highly efficient in accurately assigning the most probable population of origin of individuals of unknown origin, such as illegally confiscated birds, based on their genotype profile. Our reduced panel of highly informative genome‐wide SNPs provides a standardized, fast, and low‐cost genomic approach for conservation. Compared with the microsatellite genotyping scheme currently used (Domínguez et al. 2019), this SNP methodology offers improved genotyping quality and a more efficient workflow. Recently, diagnostic SNP panels have been successfully used for identifying the source of poached pangolins (Tinsman et al. 2023), African forest elephant (Bourgeois et al. 2018), and green abalone (Mejía‐Ruíz et al. 2023). Such approaches are easily applied in conservation programs, enabling timely management interventions to the current biodiversity crisis caused by illegal trade.

With the SNP panel developed here all confiscated birds could be assigned to their respective management unit, allowing a more efficient release of the confiscated cardinals in their most probable place of origin and thus increasing their chances of survival and reproduction. Our study indicates that most of the confiscated Yellow cardinals were assigned to MU3, with only six assigned to MU1. This finding aligns with historical data, which suggest that MU3 has historically supported more stable and numerous populations (Pessino and Tittarelli 2006), and corroborates the results of previous assignment tests based on microsatellites (Domínguez et al. 2019). However, the novel discovery of some individuals assigned to MU1 suggests that this part of the distribution is also subject to illegal wildlife trade, and might be in need of conservation strategies to protect its populations.

The wildlife trade poses a significant impact on endangered Yellow cardinals and other species, and forensic investigation using DNA‐based evidence can play a crucial role in addressing illegal trade of protected species by pinpointing the natural populations being targeted. We found a particularly interesting case of potentially international pet trade, as three cardinals sampled in Uruguay either clustered with individuals from the other extreme of the distribution in Argentina (> 1000 km), or appeared to be descendants of a cross between local and western cardinals released in Uruguay. Their genotypes suggested that these birds with unexpected grouping patterns could be a consequence of human‐mediated dispersal. Finally, our forensic approach to assign confiscated specimens to populations of origin could potentially also be applied to other species, enhancing conservation efforts by providing a reliable method to facilitate the reintegration of seized individuals into the wild.

### 4.2 Evolutionary and Conservation Implications of Intergeneric Hybridization

Investigating the extent of genetic introgression from hybridization with the Diuca finch can provide insights into how this process affects the genetic integrity of the Yellow cardinal. Such knowledge is crucial for developing strategies to minimize the potential negative impacts of hybridization and ensure the long‐term survival of the species. The Yellow cardinal is monogamous, and males maintain their territory during successive breeding periods (Domínguez et al. 2015; Beier et al. 2017; Atencio, Reboreda, and Mahler 2022). However, when there is a deviation from equal sex ratio, mostly due to a shortage of males as a consequence of the illegal pet trade market, cooperative breeding (Beier et al. 2017), polygyny (Segura et al. 2019) or hybridization with the Diuca finch (Bertonatti and Lopez‐Guerra 1997; Pessino 2006) are observed.

We used several lines of evidence to assess if hybridization occurs, whether it resulted in introgression, and to genetically confirm the existence of hybrids. Our results suggest that even though the Yellow cardinal and Diuca finch differ in morphology, have overall different biological attributes and ecological requirements, and have diverged around 4 million years ago, contemporary hybridization occasionally takes place. The three hybrids analyzed here proved to be males, being consistent with the relatively deep split between these two species and matching Haldane's (1922) rule. The latter predicts that when there is some degree of genetic incompatibility between species, it occurs more frequently in the heterogametic sex which, in birds, corresponds to females. Two of these hybrids were characterized as F1 and the remaining one as a backcross with a Yellow cardinal, proving that hybrids can be fertile. However, our results suggest that hybridization does not lead to effective introgression between species, as we detected no admixed Yellow cardinals or Diuca finches after analyzing over 100 individuals. All hybrids exhibited the mitochondrial lineage of the Yellow cardinal, supporting the hypothesis that female Yellow cardinals might hybridize with male Diuca finches, presumably as a result of a shortage of male cardinals due to illegal trade (Bertonatti and Lopez‐Guerra 1997). According to the sexual selection hypothesis for unidirectional hybridization (Wirtz 1999), females of the rarer species will eventually mate with heterospecific males from the more common species when conspecific males become scarce. In this case, the scarcity of male Yellow cardinals, driven by illegal capture and trade, may be forcing female cardinals to more frequently accept male Diuca finches as mates, highlighting the complex dynamics of hybridization driven by human activities and the importance of addressing illegal wildlife trade to maintain the natural genetic diversity and structure of affected species.

Sexually reproducing species maintain their identity from other species due to the existence of reproductive isolation mechanisms, which prevent homogenizing gene flow. Evidently, the reproductive barriers between Yellow cardinals and Diuca finches are not as strong as to prevent hybridization. However, only a few hybrids have been detected in the wild, which could be the result of high mortality rates, low biological efficiency of the hybrids, or hybridization occurring only occasionally. Because this is the first study to provide insights into this phenomenon, it is necessary to further investigate mate choice to better understand whether hybrids are a consequence of specific conditions that favor crossbreeding (e.g., scarcity of conspecific males) or, instead, the result of random events. In this context, future research should focus on increasing the sampling of sympatric regions to better assess the frequency and extent of hybridization and sex ratio, while also using playback experiments to investigate how Yellow cardinals and Diuca finches respond to interspecific songs (Fracas et al. 2023). As for the latter, Domínguez, Reboreda, and Mahler (2016) evidenced that populations of the Yellow Cardinal significantly differ in their vocalizations and that songs are more similar to those of the Diuca finch where species coexist, suggesting that hybridization may also be facilitated by song similarity.

In spite of its rather low occurrence, the fact that introgression is possible raises concerns, mainly because if it becomes more common it could threaten the genomic integrity of the endangered Yellow cardinal. Introgression can lead to the incorporation of foreign genetic material into the Yellow cardinal population, potentially diluting its unique genetic traits and adaptations. This could have several detrimental effects, such as reducing the overall fitness of the population or introducing maladaptive traits, leading to local extinctions. Alternatively, hybridization may also promote adaptive introgression if new genetic combinations are better adapted to the local environment (Hedrick 2013; Enciso‐Romero et al. 2017; Hill 2019). Therefore, ongoing monitoring and more detailed genetic studies are essential to assess the long‐term impacts of hybridization and to develop strategies for preserving the genetic integrity of this species. Conservation efforts must prioritize understanding and mitigating the factors that may facilitate hybridization to protect this endangered species from genetic threats that add to illegal trafficking and environmental transformation.

### 4.3 Final Remarks: Conservation and Evolutionary Implications

This study provides key insights into the population structure and intergeneric hybridization of the Yellow cardinal. Beyond our exploration of population structure and the establishment of three management units, we developed a reduced panel of diagnostic SNPs to reliably assign confiscated individuals in the future, translating genomics into direct management applications. Furthermore, we provide evidence that hybrids between Yellow cardinals and Diuca finches are fertile. If backcrosses with Yellow cardinals become more common, due to an increase in the overlap between the ranges of both species or an asymmetric sex ratio as a consequence of the illegal pet trade, introgression of Diuca alleles could have significant impacts on Yellow cardinal populations. Our results offer valuable genomic data that can guide conservation efforts, ensuring more effective management and conservation not only for the Yellow cardinal, but also for other species. In this context, we encourage other researchers to take a similar approach in future conservation genomic studies, in order to take advantage of new technologies to improve and enhance the efficiency of conservation and management plans.

## Author Contributions

**Marisol Domínguez:** conceptualization (lead), data curation (equal), formal analysis (equal), funding acquisition (equal), investigation (lead), methodology (equal), project administration (equal), resources (equal), software (equal), visualization (equal), writing – original draft (equal), writing – review and editing (equal). **Larissa S. Arantes:** data curation (equal), formal analysis (equal), investigation (equal), methodology (equal), software (equal), visualization (equal), writing – original draft (equal), writing – review and editing (equal). **Pablo D. Lavinia:** data curation (equal), formal analysis (equal), investigation (equal), methodology (equal), software (equal), visualization (equal), writing – original draft (equal), writing – review and editing (equal). **Nicole Bergjürgen:** investigation (supporting), resources (supporting), writing – original draft (supporting), writing – review and editing (equal). **Agustin I. Casale:** data curation (supporting), resources (supporting). **Pablo A. Fracas:** data curation (supporting), resources (supporting), writing – review and editing (equal). **Darío A. Lijtmaer:** writing – review and editing (equal). **Pablo Tubaro:** writing – review and editing (equal). **Sarah Sparmann:** methodology (equal). **Susan Mbedi:** methodology (equal). **Camila Mazzoni:** methodology (equal), supervision (equal), writing – review and editing (equal). **Bettina Mahler:** conceptualization (equal), funding acquisition (equal), investigation (equal), resources (equal), supervision (equal), writing – review and editing (equal). **Ralph Tiedemann:** conceptualization (equal), funding acquisition (equal), investigation (equal), project administration (equal), resources (equal), writing – review and editing (equal).

## Conflicts of Interest

The authors declare no conflicts of interest.

## Benefit‐Sharing Statement

Benefits from this research come from sharing data and results on public databases. The results of research have been shared with the provider communities and the broader scientific community, and the research addresses a priority concern, in this case the conservation of the endangered passerine Yellow cardinal. More broadly, our group is committed to international scientific partnerships, as well as institutional capacity building. This article reflects the ongoing international collaboration between Argentinian and German institutions.

## Supporting information


**Data S1** Supporting Information

## Data Availability

The raw RADseq sequencing data is available in Genbank with the NCBI BioProject accession number PRJNA1178452. The edited COI sequences have been deposited in Genbank with accession numbers FJ027636, FJ027941, FJ027948 and PQ496607‐29.
