# Supplementary material for: Genomics Reveal Population Structure and Intergeneric Hybridization in an Endangered South American Bird: Implications for Management and Conservation
Source: Ecol Evol. 2025 Jan 8;15(1):e70820. doi: 10.1002/ece3.70820 (PMC11707398; doi:10.1002/ece3.70820)
Supplement: Supplementary file 1 — Data S1 Supporting Information [file ECE3-15-e70820-s001.zip › SupplementaryMaterial - Dominguez_etal.docx]

**Supplemental Information for:**

**Genomics reveal population structure and intergeneric hybridization in an endangered South American bird: implications for management and conservation**

Marisol Domínguez, Larissa S. Arantes, Pablo D. Lavinia, Nicole Bergjürgen, Agustin Casale, Dario Lijtmaer, Pablo Tubaro, Sarah Sparmann, Susan Mbedi, Camila Mazzoni, Bettina Mahler, Ralph Tiedemann

**Table of Contents:**

| **Table S1** | Page 2 |
| --- | --- |
| **Table S2** | Page 3 |
| **Table S3** | Page 5 |
| **Table S4** | Page 6 |
| **Table S5** | Page 7 |
| **Table S6** | Page 8 |
| **Table S7** | Page 9 |
| **Figure S1** | Page 10 |
| **Figure S2** | Page 11 |
| **Figure S3** | Page 12 |
| **Figure S4** | Page 13 |
| **Figure S5** | Page 14 |
| **Figure S6** | Page 15 |

**Table S1.** Detail of sampling geographical coverage.

File TableS1_SamplingGeographical_Coverage_RADseq_mtDNA.xlsx

**Table S2**. Datasets used for the different analyses.

| **Dataset** | **Group** | **Number of individuals** | **Information** | **Count** | **Analysis** |
| --- | --- | --- | --- | --- | --- |
| **YC_raw**: Yellow cardinals (excluding confiscated) | Corrientes | 29 | Number of SNPs called with Stacks (Population filters: p=1 and r=0.6) | 405,866 |  |
|  | Uruguay | 7 | Number of SNPs called with Stacks (Population filters: p=1, r=0.6 and single SNP per locus) | 39,899 |  |
|  | SanLuis | 5 | Number of SNPs called with Stacks (Population filters: p=1, r=0.6 and single SNP per locus) + Minimum coverage 10 | 31,695 |  |
|  | Mendoza | 7 | Number of SNPs called with Stacks (Population filters: p=1, r=0.6 and single SNP per locus) + Minimum coverage 10 + Maximum coverage 152 (2x average coverage) | 31,166 |  |
|  | LaPampa | 12 | Number of SNPs called with Stacks (Population filters: p=1, r=0.6 and single SNP per locus) + Minimum coverage 10 + Maximum coverage 152 (2x average coverage) + Missing rate 0.8 | 12,554 | PCoA |
|  | RioNegro | 24 | Average coverage of the final filtered dataset of unliked SNPs | 85,80 |  |
|  | Total | 84 |  |  |  |
| **YC**: Yellow cardinals (excluding confiscated and 3 individuals from Uruguay) | Corrientes | 29 | Number of SNPs called with Stacks (Population filters: p=1 and r=0.6) | 405,775 |  |
|  | Uruguay | 4 | Number of SNPs called with Stacks (Population filters: p=1, r=0.6 and single SNP per locus) | 40,293 |  |
|  | SanLuis | 5 | Number of SNPs called with Stacks (Population filters: p=1, r=0.6 and single SNP per locus) + Minimum coverage 10 | 31,728 |  |
|  | Mendoza | 7 | Number of SNPs called with Stacks (Population filters: p=1, r=0.6 and single SNP per locus) + Minimum coverage 10 + Maximum coverage 151 (2x average coverage) | 31,131 |  |
|  | LaPampa | 12 | Number of SNPs called with Stacks (Population filters: p=1, r=0.6 and single SNP per locus) + Minimum coverage 10 + Maximum coverage 151 (2x average coverage) + Missing rate 0.8 | 12,591 | Genomic diversity and differentiation + STRUCTURE + DAPC + AssignmentTests |
|  | RioNegro | 24 | Number of SNPs called with Stacks (Population filters: p=1, r=0.6 and single SNP per locus) + Minimum coverage 10 + Maximum coverage 151 (2x average coverage) + Missing rate 1 | 1,581 | Contemporary migration rate estimation |
|  | Total | 81 | Average coverage of the final filtered dataset of unliked SNPs | 85,16 |  |
| **YCC**: Yellow cardinals (without 3 individuals from Uruguay) including confiscated | Corrientes | 29 | Number of SNPs called with Stacks (Population filters: p=1 and r=0.6) | 426,027 |  |
|  | Uruguay | 4 | Number of SNPs called with Stacks (Population filters: p=1 and r=0.6) + Minimum coverage 10 + Maximum coverage 159 (2x average coverage) + Missing rate 0.8 | 269,913 | SNP panel design, ASSIGNPOP, DAPC+AssignmentTests |
|  | SanLuis | 5 | Number of SNPs called with Stacks (Population filters: p=1, r=0.6 and single SNP per locus) | 38,431 |  |
|  | Mendoza | 7 | Number of SNPs called with Stacks (Population filters: p=1, r=0.6 and single SNP per locus) + Minimum coverage 10 | 30,768 |  |
|  | LaPampa | 12 | Number of SNPs called with Stacks (Population filters: p=1, r=0.6 and single SNP per locus) + Minimum coverage 10 + Maximum coverage 152 (2x average coverage) | 30,314 |  |
|  | RioNegro | 24 | Number of SNPs called with Stacks (Population filters: p=1, r=0.6 and single SNP per locus) + Minimum coverage 10 + Maximum coverage 152 (2x average coverage) + Missing rate 0.8 | 12,830 | DAPC+AssignmentTests |
|  | Confiscated | 48 | Average coverage of the final filtered dataset of unliked SNPs | 86,16 |  |
|  | Total | 129 |  |  |  |
| **YCDH**: Yellow cardinals (known origin), Diuca finches, and Hybrids | *Gubernatrix cristata* | 81 | Number of SNPs called with Stacks (Population filters: p=3 and r=0.6) | 472,694 |  |
|  | *Diuca diuca* | 27 | Number of SNPs called with Stacks (Population filters: p=3, r=0.6 and single SNP per locus) | 19,303 |  |
|  | Hybrids | 3 | Number of SNPs called with Stacks (Population filters: p=3, r=0.6 and single SNP per locus) + Minimum coverage 10 | 18,637 |  |
|  | Total | 111 | Number of SNPs called with Stacks (Population filters: p=3, r=0.6 and single SNP per locus) + Minimum coverage 10 + Maximum coverage 182 (2x average coverage) | 18,611 |  |
|  |  |  | Number of SNPs called with Stacks (Population filters: p=3, r=0.6 and single SNP per locus) + Minimum coverage 10 + Maximum coverage 182 (2x average coverage) + Missing rate 0.8 | 14,327 | PCoA+HeterozygositySTRUCTURE + HeteroHybrid index + hybrid categories |
|  |  |  | Average coverage of the final filtered dataset of unliked SNPs | 100,77 |  |

**Table S3.** SNP marker panel proposed in this study.

File TableS3_192_SNPs_outliers_K3_YC_MD.txt

**Table S4.** Reagents and cycling conditions used in the amplification of the cytochrome c oxidase subunit I mitochondrial gene. Sources are [1] Hebert *et al.* 2004, [2] Kerr et al. 2009.

| **Components** | **Volume** | **Chemical** | |
| --- | --- | --- | --- |
| Buffer | 5 µL | 5x MyTaq | |
| Forward Primer | 0.2 µM | BIRD F1 ^[1]^ | |
| Reverse Primer | 0.2 µM | BIRD R1 ^[2]^ | |
| Polymerase | 0.024 U/µL | MyTaq Polymerase (5U/µl) | |
| DNA Template | 1.8 - 4 ng/µL | DNA Template | |
| Total | 25 µL |  | |
| **Cycling Conditions** | **Time (s)** | **Temperature (°C)** | |
| Initial denaturation | 60 | 94 |  |
| Denaturation | 60 | 94 |  |
| Annealing | 40 | 45 | 5X |
| Elongation | 60 | 72 |  |
|  |  |  |  |
| Denaturation | 60 | 94 |  |
| Annealing | 40 | 51 | 35X |
| Elongation | 60 | 72 |  |
|  |  |  |  |
| Final Elongation | 250 | 72 |  |

**Table S5.** Reagents and cycling conditions used for the sex determination based on primers P2/P8. Source is [3] Griffiths *et al.* 1998.

| **Components** | **Volume** | **Chemical** | |
| --- | --- | --- | --- |
| Buffer | 5 µL | 5x MyTaq | |
| Forward Primer | 0.2 µM | P2 ^[3]^ | |
| Reverse Primer | 0.2 µM | P8 ^[3]^ | |
| Polymerase | 0.024 U/µL | MyTaq Polymerase (5U/µl) | |
| DNA Template | 3.5 – 8 ng/µL | DNA Template | |
| Total | 25 µL |  | |
| **Cycling Conditions** | **Time (s)** | **Temperature (°C)** | |
| Initial denaturation | 90 | 94 |  |
| Denaturation | 30 | 94 |  |
| Annealing | 45 | 48 | 40X |
| Elongation | 45 | 72 |  |
|  |  |  |  |
| Final Elongation | 500 | 72 |  |

**Table S6.** Genomic diversity of Yellow cardinal’s management units (MU) identified in this study. The number of samples (*N*), private alleles, polymorphic sites (S), and nucleotide diversity ($\pi$) per genetic cluster is shown.

| Management unit | *N* | Private alleles | S | π |
| --- | --- | --- | --- | --- |
| MU1 | 33 | 1088 | 7172 | 0.133 |
| MU2 | 12 | 1328 | 8069 | 0.150 |
| MU3 | 36 | 2181 | 9605 | 0.146 |

**Table S7.** Fixed SNPs between Yellow cardinals and Diuca finches.

File: TableS7_genotypes_133_fixed_SNPs_DD_YC_Hybrids.xlsx

**
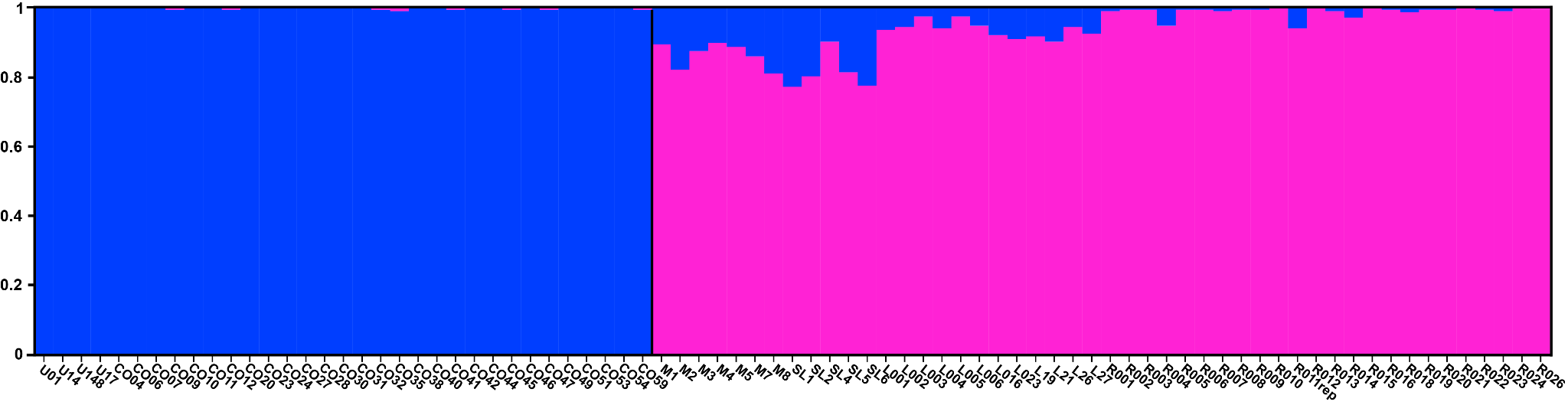
**

**Figure S1**. Structure plot (*K* = 2) for 81 wild Yellow cardinals based on ~12,500 unlinked SNPs.


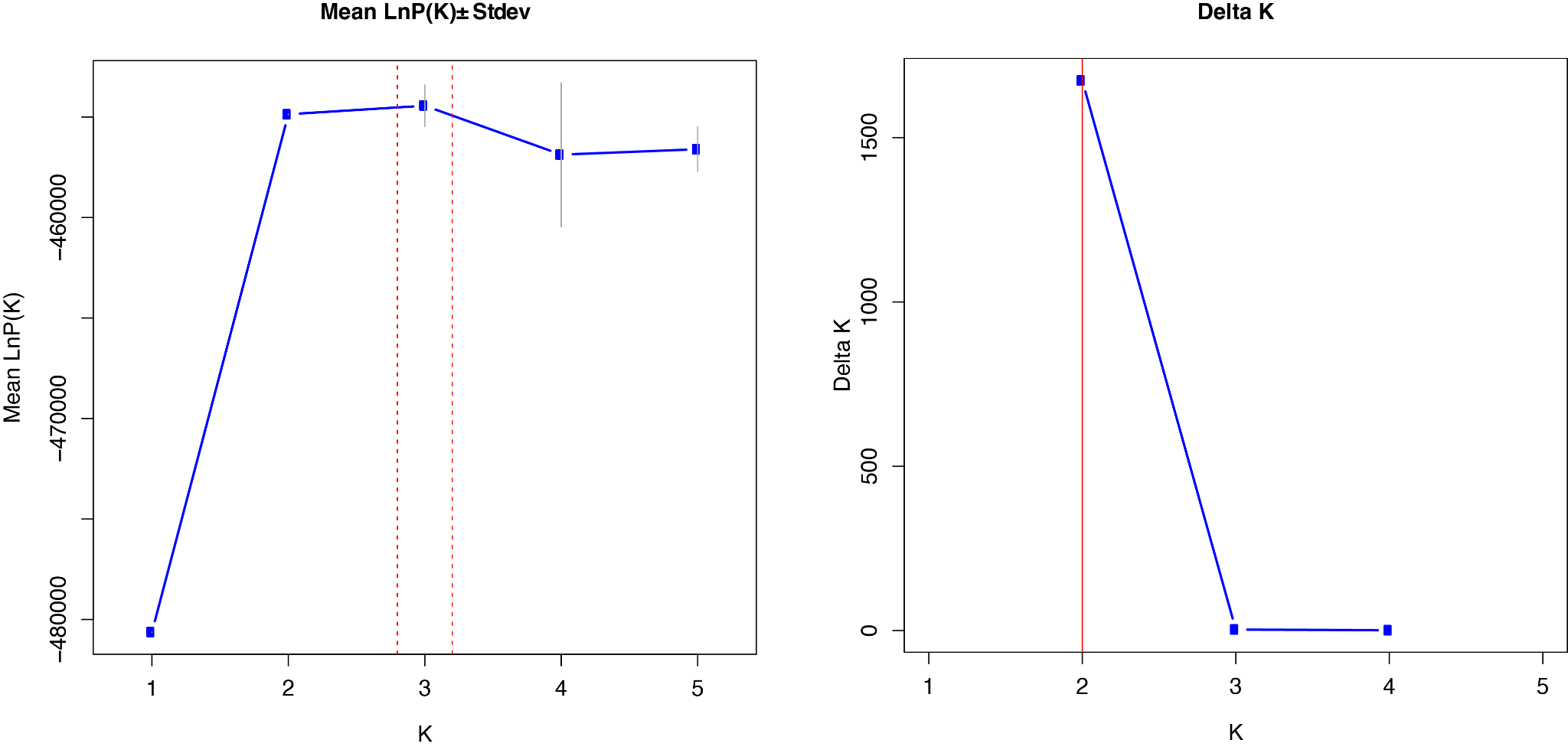


**Figure S2.** Likelihood values for *K* = 1 to *K* = 5 averaged across ten replicates per each value of *K* (left), and Evanno’s *ΔK*values (right).


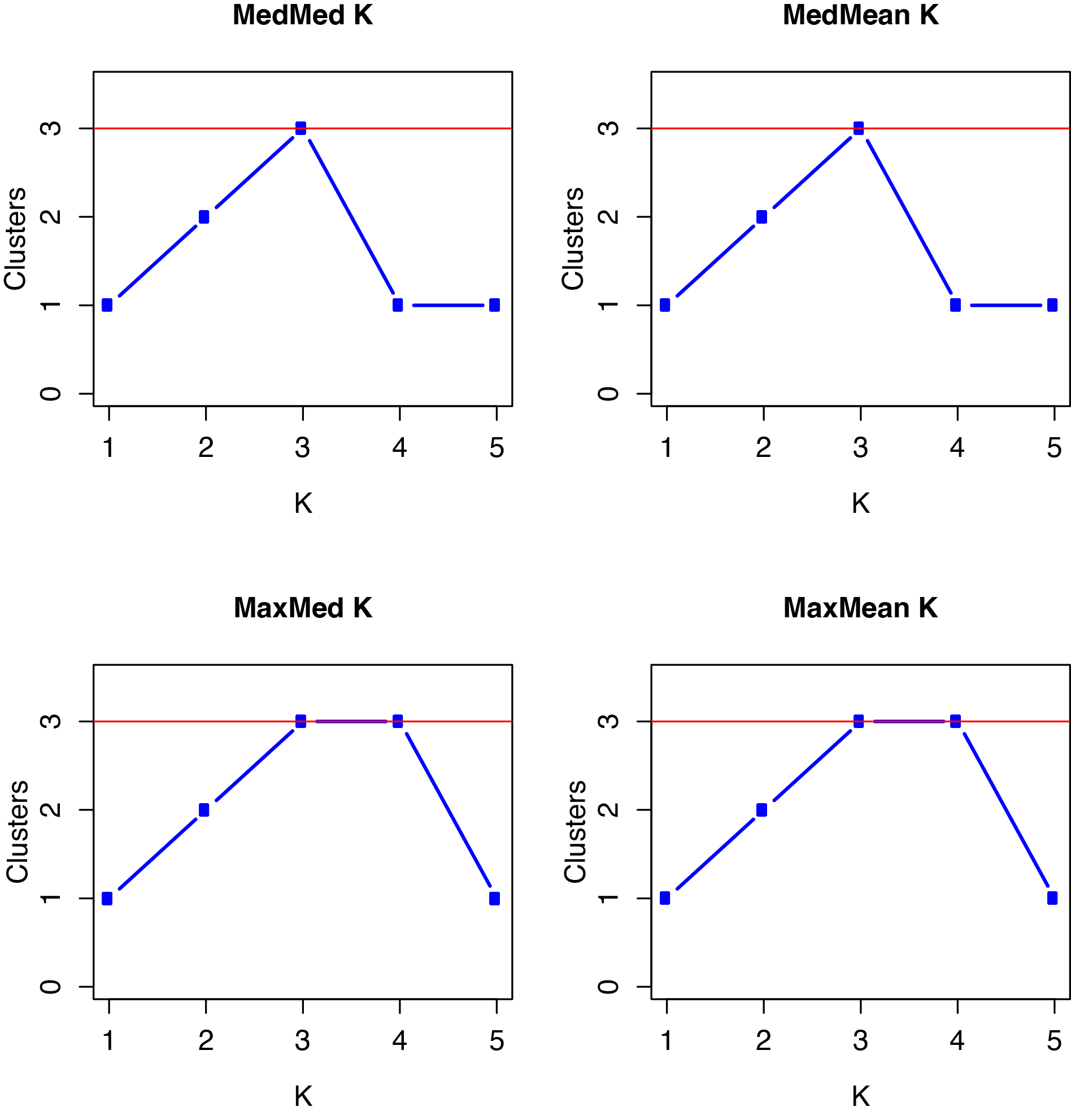


**Figure S3.** Puechmaille’s four estimators support *K* = 3 as the optimal number of genomic clusters for the Yellow cardinals.

**
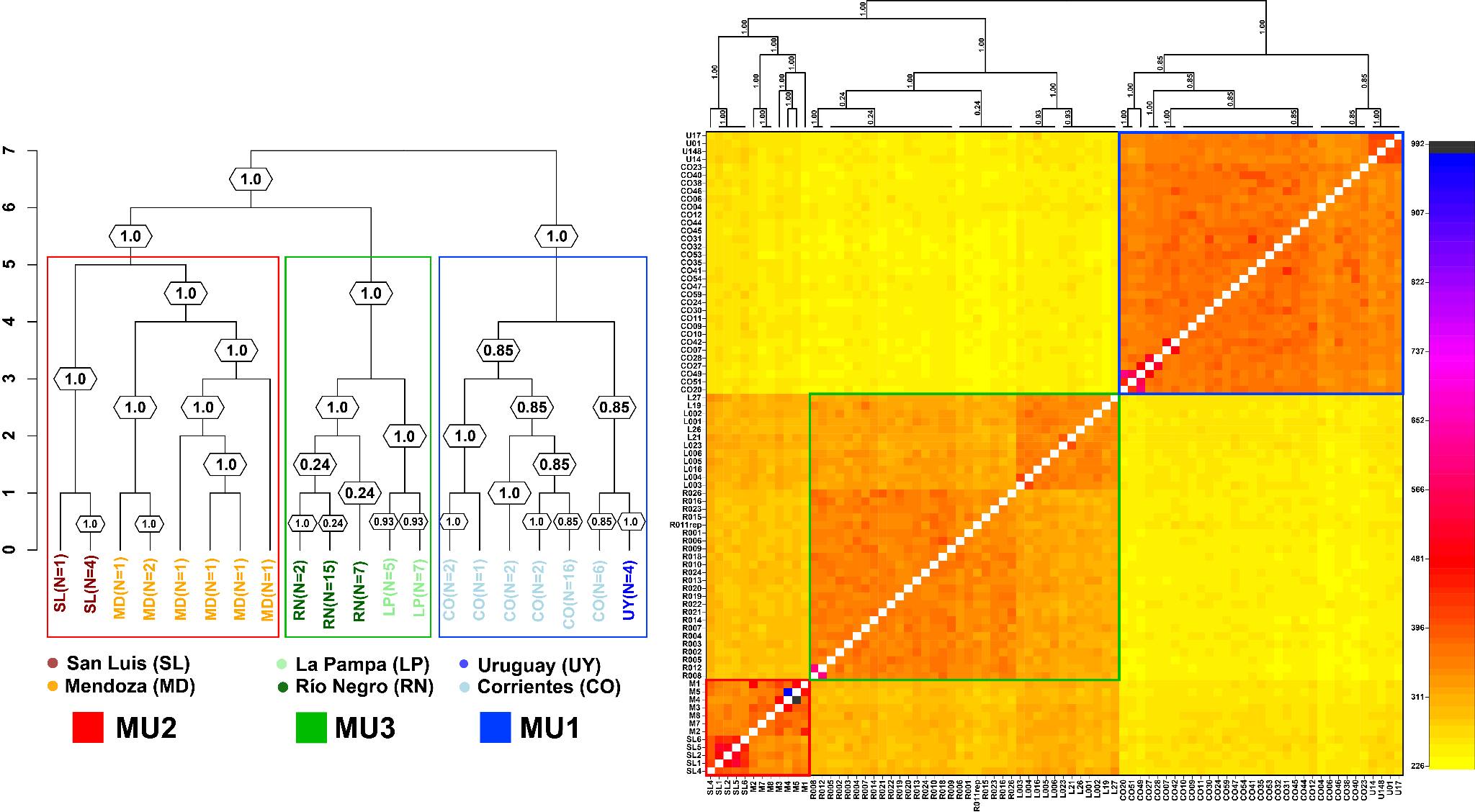
**

**Figure S4.** Results from the fineRADstructure analysis based on 133,813 SNPs from 23,522 RAD loci, retaining a maximum of 10 SNPs per RAD locus and allowing no more than 45% missing data per individual. The dendrogram on the left is not a phylogenetic hypothesis, but a rather simple MAP tree (Lawson *et al.* 2012) depicting relationships among groups based on genomic similarity. Numbers on branches indicate posterior population assignment probabilities, and tip labels indicate sampling localities and the number of individuals (N). To the right, the coancestry matrix indicates the level of coancestry among all individuals, which appear listed in the left and bottom margins of the matrix. The estimated coancestry scale appears to the right of the matrix (highest levels of coancestry are indicated by black, blue and purple colours), and the dendrogram appears at the tops. Management units (MU1, MU2 and MU3) as discussed in the main text are highlighted in both figures.

**
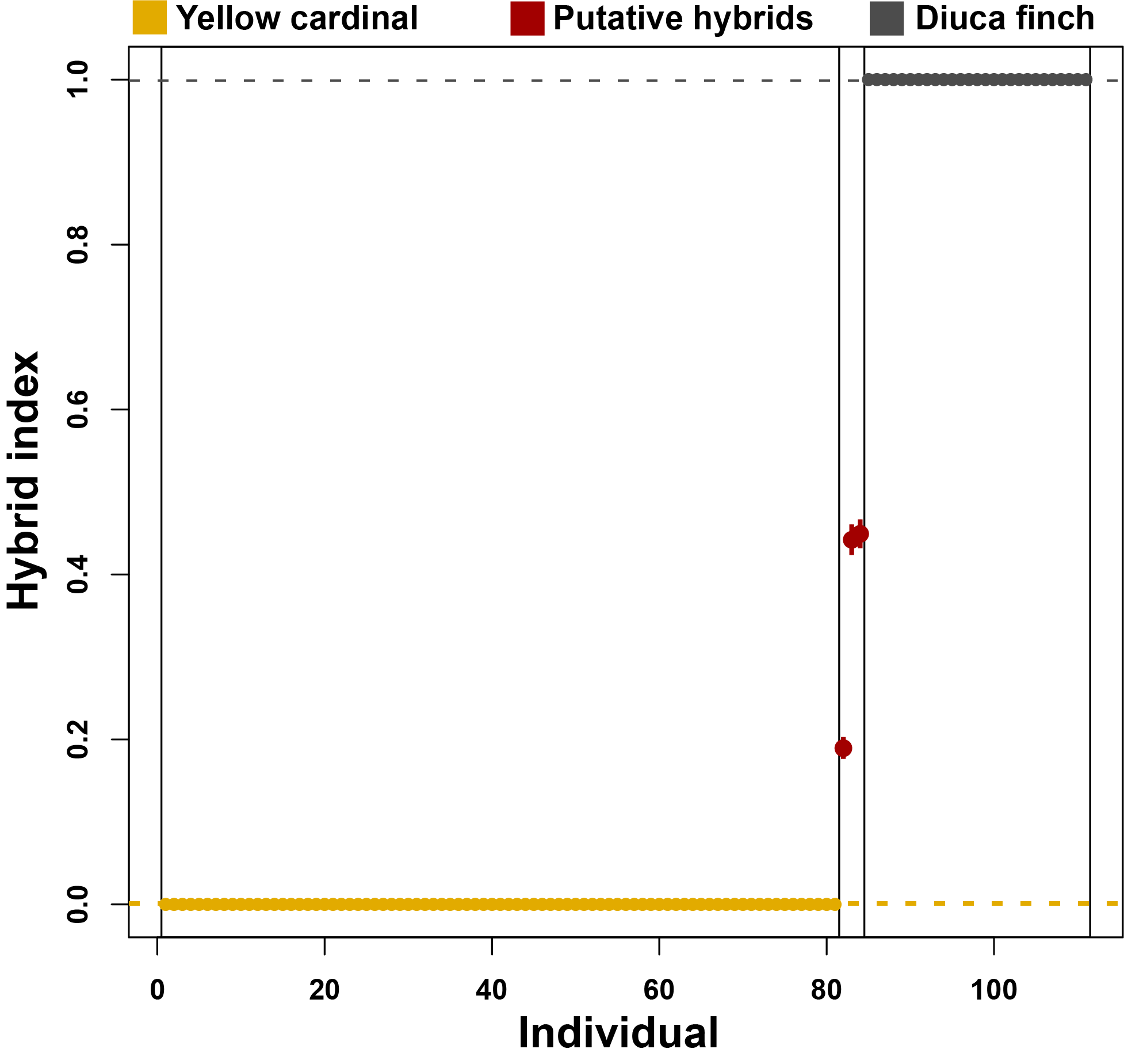
**

**Figure S5.** Genomic hybrid index and 95% confidence interval based on 14,327 SNPs for each of the 111 individuals from the YCDH dataset (including wild Yellow cardinals, Diuca finches, and putative hybrids), showing the proportion of alleles inherited from reference parental species as *G. cristata* (HI = 0) and *D. diuca* (HI = 1).

**
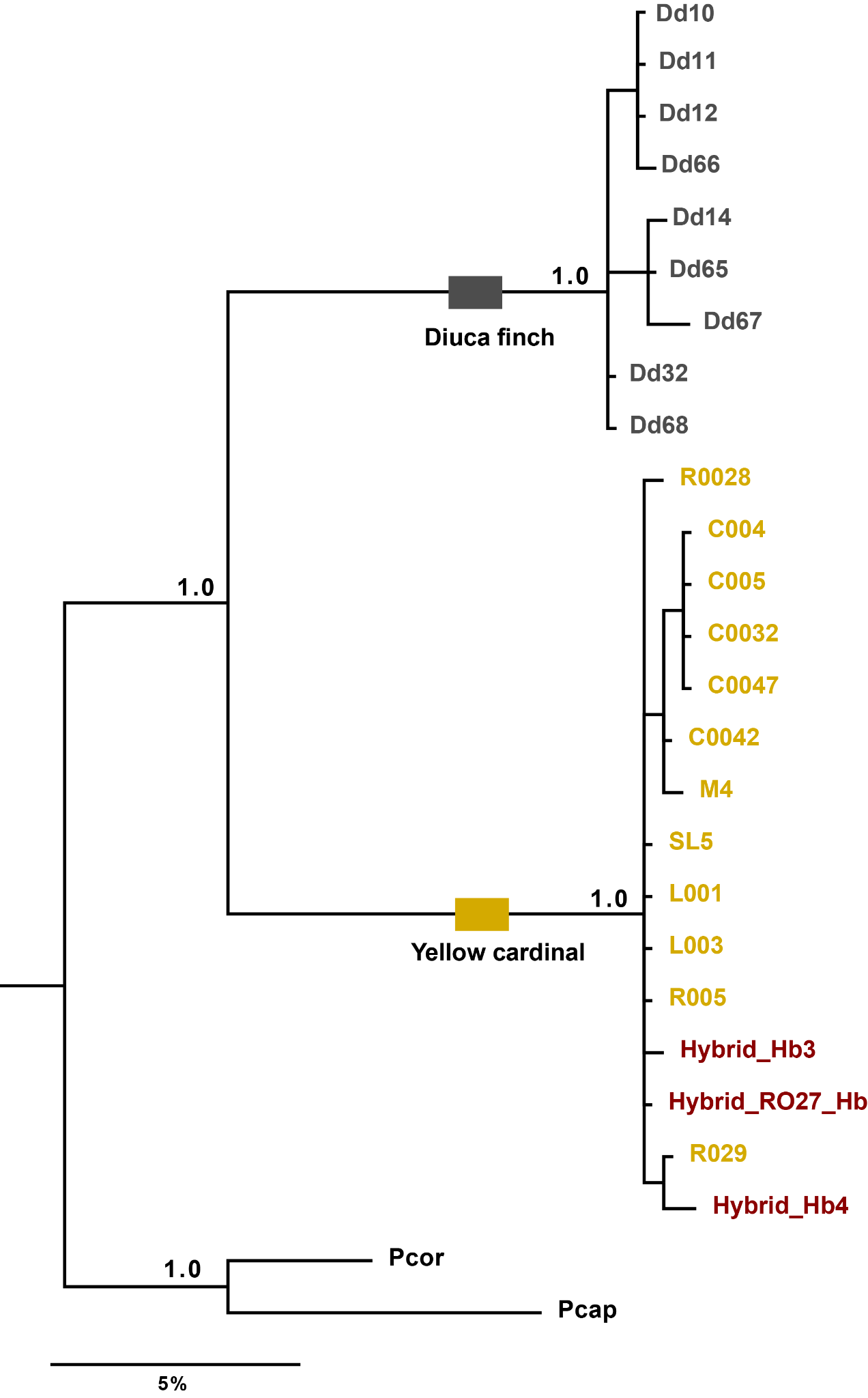
**

**Figure S6.** Bayesian majority rule consensus gene tree based on 694 bp of the mitochondrial gene COI. Numbers above the branches indicate node support (Bayesian posterior probability).
